# Adaptive Sensing Data Augmentation for Drones Using Attention-Based GAN

**DOI:** 10.3390/s24165451

**Published:** 2024-08-22

**Authors:** Namkyung Yoon, Kiseok Kim, Sangmin Lee, Jin Hyoung Bai, Hwangnam Kim

**Affiliations:** 1 School of Electrical Engineering, Korea University, Seoul 02841, Republic of Korea; nkyoon93@korea.ac.kr (N.Y.); kisuk528@korea.ac.kr (K.K.); lsm5505@korea.ac.kr (S.L.); 2 Digital Convergence Department, KEPCO E&C, Gimcheon 39660, Republic of Korea; jhbai@kepco-enc.com

**Keywords:** drone, deep learning, generative adversarial network, attention mechanism

## Abstract

Drones have become essential tools across various industries due to their ability to provide real-time data and perform automated tasks. However, integrating multiple sensors on a single drone poses challenges such as payload limitations and data management issues. This paper proposes a comprehensive system that leverages advanced deep learning techniques, specifically an attention-based generative adversarial network (GAN), to address data scarcity in drone-collected time-series sensor data. By adjusting sensing frequency based on operational conditions while maintaining data resolution, our system ensures consistent and high-quality data collection. The *spatiotemporal* The attention mechanism within the GAN enhances the generation of synthetic data, filling gaps caused by reduced sensing frequency with realistic data. This approach improves the efficiency and performance of various applications, such as precision agriculture, environmental monitoring, and surveillance. The experimental results demonstrated the effectiveness of our methodology in extending the operational range and duration of drones and providing reliable augmented data utilizing a variety of evaluation metrics. Furthermore, the superior performance of the proposed system was verified by comparing it with various comparative GAN models.

## 1. Introduction

Drones have become indispensable tools across a wide range of industries, including agriculture, logistics, surveillance, and environmental monitoring [1,2,3,4]. Their ability to provide real-time data from hard-to-reach areas and perform automated tasks has made them invaluable for various applications. In agriculture, drones can monitor crop health and optimize resource use [5,6]. In logistics, they can deliver goods quickly and efficiently [7]. Surveillance drones enhance security by providing aerial views in real time, while environmental monitoring drones track changes in ecosystems, enabling timely interventions [8,9].

As the demand for drones increases, so does the need to equip them with a diverse array of sensors. These sensors enable drones to perform more complex tasks and improve their overall performance by collecting detailed and multifaceted data. For instance, drones equipped with cameras, thermal sensors, LIDARs, and various environmental sensors can provide comprehensive insights that are usually difficult for drones to provide for sensing [10]. The integration of multiple sensors can significantly enhance the capabilities of drones, allowing them to undertake a broader range of missions with higher precision and efficiency.

However, a critical challenge arises with the integration of numerous sensors in a single drone [11]. The payload capacity of drones is limited, and adding too many sensors can lead to significant network burden and data management issues [12]. High-frequency data collection from multiple sensors can overwhelm the onboard processing capabilities and network bandwidth, leading to potential inefficiencies and performance bottlenecks. The high data rate required for real-time processing and transmission can cause delays, increase power consumption, and limit the drone’s operational range and duration [13]. Selectively reducing the sensing frequency can mitigate the network burden. However, this approach can lead to data scarcity, which might negatively impact the performance of applications relying on comprehensive sensor data [14,15,16,17]. Given these constraints, it is crucial to explore innovative solutions that can maximize the utility of sensor data without exacerbating the physical and computational load on drones.

Various methods have been proposed to address these challenges. One potential solution lies in data augmentation techniques that can enhance sensor data without increasing the physical sensor load [18]. One of the most promising augmentation approaches is data augmentation using generative adversarial networks (GAN) [19]. In addition to GAN, other augmentation techniques such as variational autoencoders (VAEs) [20] and recurrent neural networks (RNNs) [21]-based models such as sequence-to-sequence (Seq2Seq) [22] have also shown potential in generating high-quality synthetic data. VAEs can generate synthetic data by learning the underlying distribution of the data and then sampling from this distribution to produce new data points [23]. However, VAEs often produce blurrier and less detailed outputs compared to GANs, making them less suitable for applications that require high-fidelity data augmentation. Despite their effectiveness, Seq2Seq models can struggle to capture the complex and high-dimensional dependencies present in drone sensor data. Seq2Seq models, which are a type of RNN, are particularly effective in generating sequential data by learning sequence patterns and generating new sequences that follow the same patterns [24]. Despite their effectiveness, Seq2Seq models may struggle to capture the complex and high-dimensional dependencies present in drone sensor data.

However, it has been studied that these generative techniques have challenges for the characteristics of large deviations of multivariate time-series data, such as drone sensor data shown in the following sections [25,26]. Therefore, we propose a new generative model that can generate realistic synthetic sequences based on GAN that can generate synthetic data very similar to real data by learning the patterns and distributions of the data [27,28]. Furthermore, we aim for appropriate models for the diverse and evolving properties of sensor data in drone applications by enabling continuous improvement and adaptation through the competitive training process of GAN.

In this paper, we propose a comprehensive system designed to address the data scarcity issue in drone-collected time-series sensor data. Our system integrates advanced deep learning techniques with novel attention mechanisms to enhance the generation of synthetic data. Our primary contributions are as follows:

- **Integration of Advanced Deep Learning Techniques and a Novel** ***Spatiotemporal*** **Attention Mechanism:** We leverage cutting-edge methods to enhance the generation of synthetic data, providing a robust solution for augmenting time-series data. Our novel *spatiotemporal* attention mechanism excels at capturing both spatial and temporal dependencies across sequences, significantly improving the performance of deep learning models on drone-collected sensor data. By addressing the intricate relationships between spatial and temporal elements, this mechanism ensures a more comprehensive understanding of the data, leading to better predictive accuracy and model reliability [29]. Furthermore, this mechanism is adaptable to various data dimensions and types, making it versatile for different applications. It also facilitates the effective integration of partial convolution layers within our proposed GAN architecture, which helps in handling missing data and irregular time-series patterns, thereby enhancing the overall robustness and functionality of the system.
- **Efficiency and Accuracy in Data Augmentation:** Our system enables efficient and accurate augmentation of sensor data, allowing for the collection of sensor data at longer intervals without compromising the quality or quantity of the data. By reducing the sensing frequency, we significantly alleviate network and power burdens, which are critical constraints in drone operations. This approach ensures that the drones can operate for extended periods and cover larger areas without frequent returns for data offloading or battery recharges. Consequently, drones can sustain high performance in various applications such as precision agriculture, environmental monitoring, and surveillance by lowering the sensing frequency and maintaining data volume. The augmented data maintain high fidelity to real-world conditions, ensuring that applications relying on comprehensive sensor data can continue to perform optimally [30].

The rest of this paper is structured as follows: Section 2 reviews existing GAN models and attention algorithms. In Section 3, we elaborate on the architecture of our proposed GAN and the *spatiotemporal* attention mechanism. Section 4 presents the experimental results, showcasing the effectiveness of our attention mechanism and the augmented data generated by our GAN. Finally, Section 5 summarizes the paper and outlines possible directions for future work.

## 2. Preliminary

### 2.1. Generative Adversarial Networks

Generative adversarial networks (GANs) comprise two symmetrical deep learning architectures that engage in an adversarial learning process. This innovative framework, introduced by Goodfellow et al. in 2014, has revolutionized the field of generative modeling [19]. The system involves a generative neural network that creates synthetic data and a discriminative neural network that differentiates real data from synthetically generated data. The training process involves an iterative competition between the two networks: the generative network aims to generate data that the discriminative network cannot distinguish from real data, while the discriminative network endeavors to accurately identify synthetic data. This form of unsupervised learning, known as adversarial training, does not require data labeling, which makes it highly efficient for various applications.

In a typical GAN configuration, the generative network uses random noise as input to create synthetic data. The synthetic data are then fed into the discriminative network. The generative network amplifies the feature map, generating high-dimensional data such as images, audio, and sequences from low-dimensional noise. However, the discriminative network, constructed using a traditional artificial neural network structure, diminishes the feature map, classifying the data as real or synthetic. The interplay between these networks can be likened to a cat-and-mouse game where the generator improves its output to deceive the discriminator, while the discriminator gets better at detecting fake data [27].

Goodfellow et al. [19] defines generative adversarial nets as a minimax game with a value function V(G,D), represented by the equation:

(1) $$\min_{G}\left.\max_{D}\right.V\left(D,G\right)=Ex∼pdata(x)\left[\log D\left(x\right)\right]+Ez∼pz(z)\left[\log\left(1-D\left(G\left(z\right)\right)\right)\right].$$

Here, *x* represents the real data sampled from the data distribution $p_{data}\left(x\right)$, and *z* represents the noise vector sampled from the prior distribution $p_{z}\left(z\right)$. The generator *G* aims to minimize this objective against the discriminator *D* that tries to maximize it. This formulation ensures that the generator produces increasingly realistic data over time as it learns to better mimic the true data distribution.

Over the years, several variants of GANs have been developed to address specific challenges and improve performance.

- **Conditional GANs (cGANs):** These incorporate auxiliary information, such as class labels, to generate class-specific data, making the models more controllable and versatile [31].
- **Wasserstein GANs (WGANs):** These use the Wasserstein distance as a loss function to stabilize training and mitigate issues such as mode collapse [32].
- **CycleGANs:** These are designed for image-to-image translation tasks without requiring paired training examples, enabling applications like style transfer and domain adaptation [33].
- **StyleGANs:** These have advanced the field of high-resolution image synthesis, particularly in generating highly realistic human faces [34].

GANs are also used for data augmentation, where synthetic data help train machine learning models when real data are scarce or expensive to acquire [25,35]. In various fields, GANs are utilized to improve the performance of applications, and in natural language processing, GANs contribute to text generation and language translation tasks [36].

### 2.2. Attention Mechanism

Artificial intelligence has evolved to learn and apply various forms of data. In particular, RNNs are deep learning models that excel at learning sequentially listed time-series data, such as language translation [37]. However, RNNs face significant challenges when dealing with long sequences due to the bottleneck phenomenon. This issue arises because existing RNNs sequentially accumulate input data in a hidden state, and when all data are entered, the first data points compressed in the hidden state may not be properly used due to the vanishing gradient problem [38]. This bottleneck phenomenon can cause performance degradation because the deep learning model cannot properly utilize important data that may have been introduced early in the sequence.

To address this limitation, the attention mechanism was developed. Attention mechanisms compute which parts of the dataset are most relevant at each step of the processing, allowing the model to focus on important information dynamically [29,39]. This approach mitigates the bottleneck problem by enabling the model to reference all parts of the input sequence directly, regardless of their position. Attention mechanisms have become integral to many advanced neural network architectures, particularly in Natural Language Processing (NLP) and computer vision tasks [40].

Attention can be broadly categorized into several types:

- **Global Attention:** Considers the entire input sequence when computing attention scores, providing a comprehensive context [41].
- **Local Attention:** Focuses on a smaller, fixed-size window around a particular point in the input sequence, which is useful for tasks where locality is important [41].
- **Self-Attention:** Also known as intra-attention, this mechanism relates different positions of the same sequence to compute a representation of the sequence. It is a crucial component of Transformer models, which have set new benchmarks in NLP tasks [29].

In deep learning models, the attention mechanism computes attention scores to focus on crucial parts of the dataset, enabling the model to give more weight to significant inputs and less weight to less relevant data [37]. The detailed implementation of the attention mechanism is further described in the neural network structure of Section 3.

Additionally, the attention mechanism addresses the chronic problem in existing deep learning models known as the black box problem [42]. Traditional deep learning models often operate in an opaque manner, making it difficult to understand how specific inputs influence the model’s decisions. Applying the attention mechanism can help visualize and interpret which parts of the input data the model is focusing on, thus providing insights into the model’s decision-making process [40].

### 2.3. Quantile Transformer Preprocessing

The quantile transformer is a widely used technique in data preprocessing that transforms the distribution of data to a specified target distribution. This is particularly useful when the data do not meet certain assumptions, aiding in enhancing the performance of statistical analyses or machine learning models. The quantile transformer estimates the cumulative distribution function (CDF) of the original data and uses it to map each value to the corresponding quantile of the target distribution. Through this process, the data is transformed into a new distribution, making the quantile transformer a valuable tool for improving model performance by altering the data’s distribution. It is especially useful when dealing with non-normal distributions, as it can mitigate the impact of outliers and enhance the generalization performance of the model. However, careful consideration should be given to the computational cost and potential information loss that may occur during the transformation process.

## 3. System Design

### 3.1. System Overview

**Figure 1:**
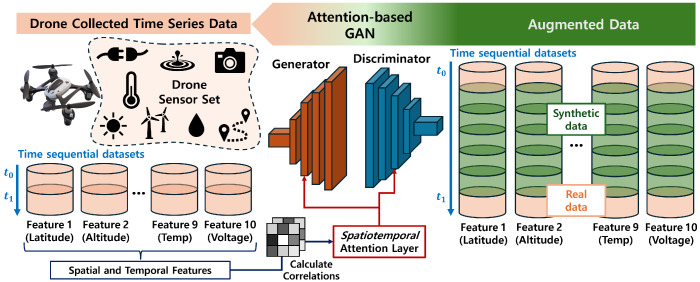
System Overview: Attention-based GAN for time-series sensor data augmentation.

In this research, we propose a methodology that leverages drones equipped with multiple sensors to collect data in a manner that allows for adaptive sensing frequencies based on operational conditions while maintaining consistent data resolution. Figure 1 shows an overview of the proposed system. Drones are outfitted with a variety of sensors tailored to the data requirements of different applications, capturing extensive time-series data. Rather than limiting the sensors to the minimum necessary, utilizing the maximum number of sensors within the capacity of a drone can significantly enhance the performance of various applications. The capabilities and performance of drones are influenced by factors such as climate, geography, and mission objectives. Therefore, it is essential to adjust system performance flexibly according to the situation to ensure efficient drone operations. However, changing the performance settings of the system can impact the quality of the sensor outputs, leading to inconsistent data collection. These inconsistencies can hinder the ability to consistently collect high-quality data and can lead to data scarcity issues.

To address these challenges, we utilize a *spatiotemporal* attention-based generative model. Our model is designed to fill the gap between sensor data arising from variations in sensing intervals with synthetic data very similar to real data. By leveraging the detailed relationships within the sensor data, the proposed model accurately captures complex interactions to produce realistic synthetic datasets, focusing on the inherent dynamics of drone operation. The *spatiotemporal* attention mechanism specifically hones in on the critical correlations and patterns within the time-series data, ensuring that the generated synthetic data maintains the integrity and consistency of real-world sensor readings. This approach takes into account the complex and rapidly changing environmental and operational parameters, effectively capturing the nuances and variability inherent in drone sensor data. The proposed model allows for continuous, high-resolution data to be provided even if the sensing frequency is flexibly adjusted according to operational needs, as well as addressing data shortages.

### 3.2. Exploratory Data Analysis for Drone Sensor

In this section, we present an exploratory data analysis (EDA) of the drone sensor data collected from various campaigns conducted in Spain and Italy. The data provide a diverse set of conditions and locations, which are essential to generating robust synthetic data, as shown in Table 1.

**Figure 2:**
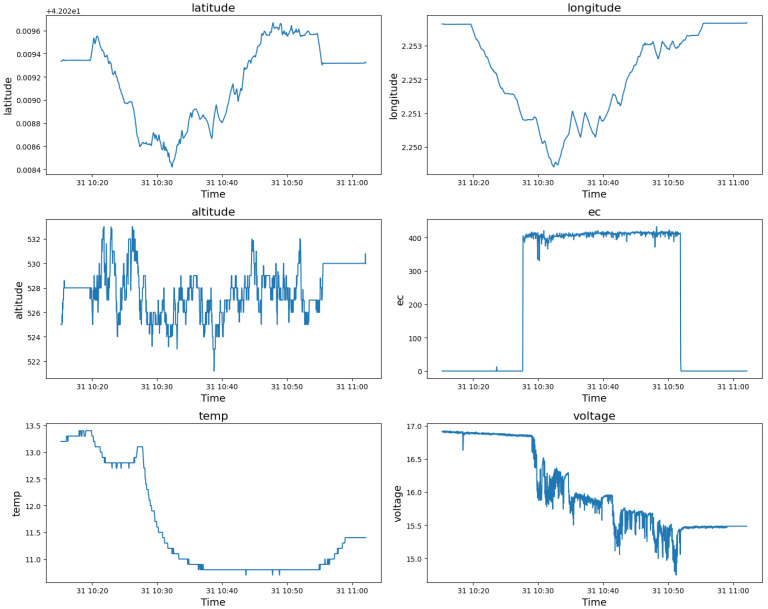
Time Series Plots of Drone Sensor Data for ESP2 in Section 3.2.

The data include multiple sensor readings such as latitude, longitude, altitude, electrical conductivity (EC), temperature (temp), and voltage. We perform a series of visualizations showing the temporal variations and distributions of the main variables, latitude, longitude, altitude, EC, temperature, and voltage, to understand the data. The time series plots represent time-dependent fluctuations and show patterns and anomalies of sensor values, as shown in Figure 2. All data possess temporal continuity, allowing reasonable inference of specific values based on preceding and subsequent values at a given point in time. We utilize various neural network techniques to analyze the temporal continuity of each feature within the dataset and the correlations between these features. Based on this analysis, we generate synthetic data that closely resemble real-world data.

**Figure 3:**
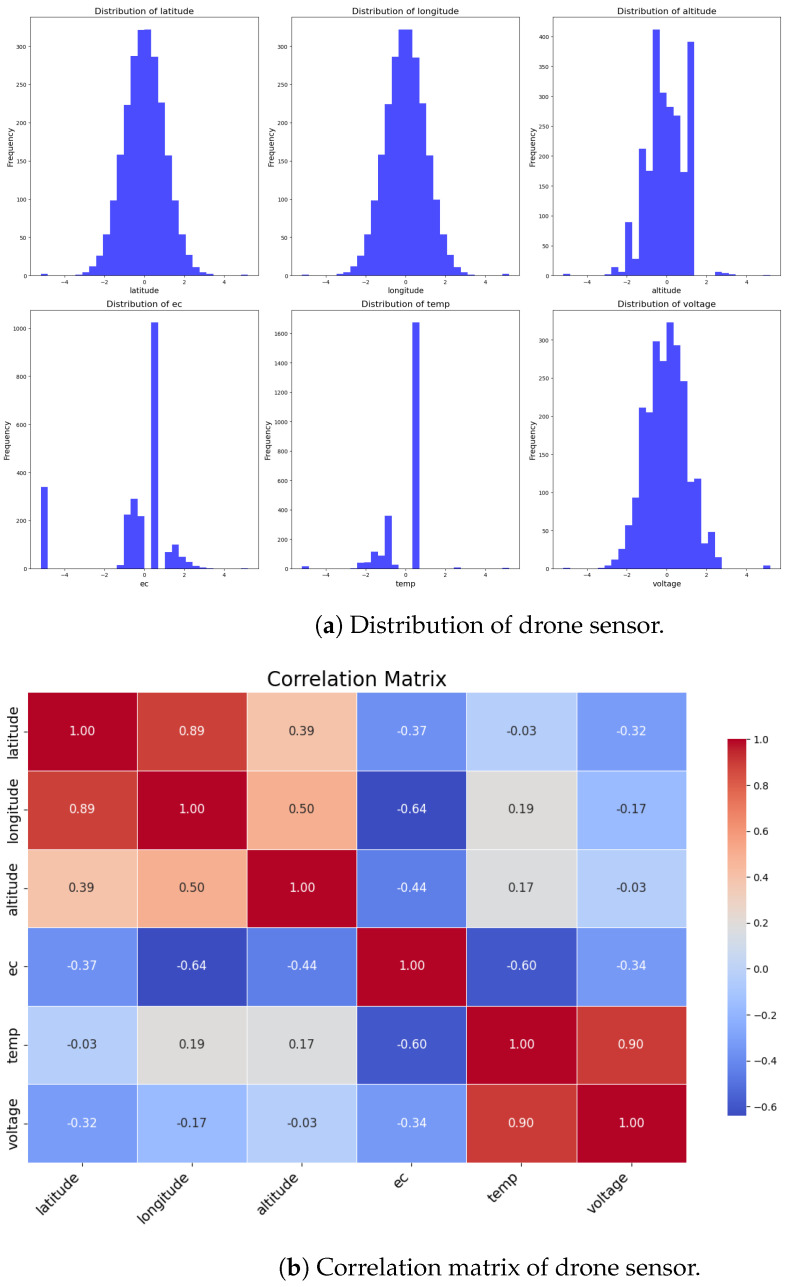
Exploratory data analysis on drone data for ESP2.

In addition, we show the distribution characteristics of each variable through the histogram and show the range and frequency of sensor readings as shown in Figure 3a. These histograms are used to visually compare the quality of the synthetic data generated by our generated model in the subsequent performance evaluation section. There is a previous study showing that the transformer-based generation model, which is widely used, including ChatGPT, is suitable for datasets with Zipfian distribution and burstiness characteristics [43]. However, the histogram like the figure shows that the data from our study do not have Zipfian distribution and burstiness characteristics. Therefore, we propose a generation technique using GAN based on previous studies related to multivariate time series data. We also use correlation matrices such as Figure 3b to identify positive correlations, negative correlations, and correlation strengths to highlight the relationships between different variables. The correlation matrix in Figure 3b is calculated using Pearson correlation coefficients, which quantify the linear relationships between pairs of variables by dividing the covariance of the variables by the product of standard deviations. This provides a comprehensive view of how each pair of variables interacts, with values from −1 representing perfect negative correlations to 1 representing perfect positive correlations and 0 indicating no linear correlation. Through this, we propose an attention mechanism focusing on correlation variables to seamlessly learn and generate realistic synthetic time-series sensor data. We intend to resolve the lack of data in drone operation by generating synthetic time-series sensor data and contribute to the advancement of autonomous drone systems by improving data generation techniques.

In the next section, we propose a novel approach to generating realistic drone data using GAN with novel attention mechanisms. Moreover, we design models that capture complex relationships between different sensor readings by leveraging insights from exploratory data analysis.

### 3.3. Spatiotemporal Attention Mechanism

We propose a novel *spatiotemporal* attention mechanism based on EDA, aiming to accurately reflect the complex interactions within drone sensor readings to generate realistic synthetic datasets. Utilizing these relationships, the *spatiotemporal* attention mechanism focuses on the inherent dynamics of drone operation, focusing on important relationships between different time-series sensor data, especially correlations between rapidly changing temperatures, latitude, longitude, altitude, voltage, and EC, do, heading of drones. Algorithm 1 shows the process of the proposed spatiotemporal attention mechanism. Multivariate features in time series data are correlation-based, especially when spatial factors such as latitude and longitude change rapidly around their relationships with other sensor elements.
**Algorithm 1** Algorithm of *spatiotemporal* attention mechanism**Require:** Input matrix $x\in R^{n\times d}$
**Ensure:** Output
  1:Initialize weight matrices $W_{\mathrm{pos}},W_{\mathrm{neg}}\in R^{d\times d}$  2:Initialize bias vectors $b_{\mathrm{pos}},b_{\mathrm{neg}}\in R^{d}$  3:Initialize attention vectors $u_{\mathrm{neg}},u_{\mathrm{corr}}\in R^{d}$  4:Compute positive correlation attention: $u_{\mathrm{pos}}\leftarrow \tanh\left(xW_{\mathrm{pos}}+b_{\mathrm{pos}}\right)$  5:Compute attention weights for positive correlations: $a_{\mathrm{pos}}\leftarrow \frac{\exp(u_{\mathrm{pos}}C_{\mathrm{pos}})}{\sum_{j=1}^{n}\exp\left(u_{\mathrm{pos},j}C_{\mathrm{pos},j}\right)}$  6:Compute output for positive correlations: ${\mathrm{output}}_{\mathrm{pos}}\leftarrow x⊙a_{\mathrm{pos}}$  7:Compute negative correlation attention: $u_{\mathrm{neg}}\leftarrow \tanh\left(xW_{\mathrm{neg}}+b_{\mathrm{neg}}\right)$  8:Compute attention weights for negative correlations: $a_{\mathrm{neg}}\leftarrow \frac{\exp(u_{\mathrm{neg}}C_{\mathrm{neg}})}{\sum_{j=1}^{n}\exp\left(u_{\mathrm{neg},j}C_{\mathrm{neg},j}\right)}$  9:Compute output for negative correlations: ${\mathrm{output}}_{\mathrm{neg}}\leftarrow x⊙a_{\mathrm{neg}}$10:Combine outputs: $\mathrm{output}\leftarrow \sum_{i=1}^{n}{\mathrm{output}}_{\mathrm{pos},i}+\sum_{i=1}^{n}{\mathrm{output}}_{\mathrm{neg},i}$11:**return** **output**


The *spatiotemporal* attention mechanism is implemented as a custom layer within our GAN architecture. This *spatiotemporal* attention mechanism is designed to focus on important correlations between different time-series sensor data. Let $x\in R^{n\times d}$ be the input matrix, where *n* is the number of time steps and *d* is the dimensionality of the input features. The *spatiotemporal* attention mechanism focuses on two main aspects, which appear to be positive and negative correlations of time-series sensor data.

To further enhance the attention mechanism, we incorporate the correlation calculation. The correlation matrix C between different time-series sensor data is defined as follows:

(2) $$u_{\mathrm{pos}}=\tanh\left(xW_{\mathrm{pos}}+b_{\mathrm{pos}}\right),$$

(3) $$a_{\mathrm{pos}}=\frac{\exp(u_{\mathrm{pos}}C_{\mathrm{pos}})}{\sum_{j=1}^{n}\exp\left(u_{\mathrm{pos},j}C_{\mathrm{pos},j}\right)}$$

(4) $${\mathrm{output}}_{\mathrm{pos}}=x⊙a_{\mathrm{pos}},$$

where $W_{\mathrm{pos}}\in R^{d\times d}$, $b_{\mathrm{pos}}\in R^{d}$ and $u_{\mathrm{pos}}\in R^{n\times d}$ are trainable parameters, and ⊙ represents element-wise multiplication used to adjust the importance of each feature in the input [29].

Similarly, for the attention on negative correlations, we define as follows:

(5) $$u_{\mathrm{neg}}=\tanh\left(xW_{\mathrm{neg}}+b_{\mathrm{neg}}\right),$$

(6) $$a_{\mathrm{neg}}=\frac{\exp(u_{\mathrm{neg}}C_{\mathrm{neg}})}{\sum_{j=1}^{n}\exp\left(u_{\mathrm{neg},j}C_{\mathrm{neg},j}\right)},$$

(7) $${\mathrm{output}}_{\mathrm{neg}}=x⊙a_{\mathrm{neg}},$$

where $W_{\mathrm{neg}}\in R^{d\times d}$, $b_{\mathrm{neg}}\in R^{d}$, and $u_{\mathrm{neg}}\in R^{n\times d}$ are trainable parameters.

Considering the relationships derived from the EDA as shown in Figure 2, it was found that significant changes in latitude and longitude have strong positive correlations of time-series sensor data with temperature and voltage and a negative correlation with EC. On the other hand, changes in altitude exhibit relatively weak correlations of time-series sensor data with these variables. These insights guided the design of our *spatiotemporal* attention mechanism to focus on these specific relationships, ensuring that the generated data closely mimics the real-world dynamics of drone operations.

The final output of the *spatiotemporal* attention mechanism is a combination of the attention outputs as follows:

(8) $$\mathrm{output}=\sum_{i=1}^{n}{\mathrm{output}}_{\mathrm{pos},i}+\sum_{i=1}^{n}{\mathrm{output}}_{\mathrm{neg},i}.$$

We aim to use the *spatiotemporal* attention mechanism to effectively capture important correlations between different time-series sensor data in our model.

### 3.4. Deep Learning Architecture

**Figure 4:**
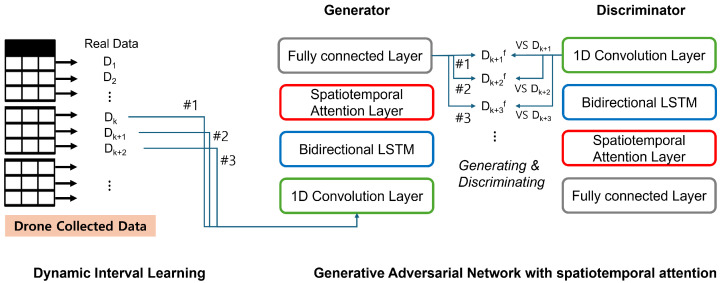
GAN architecture with a spatiotemporal attenion mechanism.

We aim to generate realistic synthetic data over GAN that accurately captures the complex dependencies within the drone sensor readings. Thus, we design an elaborate GAN architecture with a spatiotemporal attention mechanism shown in Figure 4.

First, as shown in Figure 2, quantile transformer preprocessing is applied to stabilize model learning by normalizing the distribution of voltage data and to improve the generalization performance of the model by reducing the influence of extreme values. This aims to better capture complex patterns, as quantum transformers can be effective for time series data that can have distributions, especially voltage data, through nonlinear transformations.

In this paper, the generator consists of several 1D convolution layers and a bidirectional LSTM layer to capture time dependencies to generate realistic synthetic data, followed by a spatiotemporal attention mechanism. This design ensures that both global time patterns and local sequence features are effectively captured. The final output layer uses a linear activation function to generate voltage data.

Additionally, we propose a generator loss function including mean absolute error (MAE) that ensures that the synthetic voltage value approaches the actual value to improve the fidelity of the synthetic data. The mean squared error (MSE) component makes the model more sensitive to larger errors, improving accuracy. In addition, to improve the smoothness of the synthetic data, the gradient penalty term is integrated as follows:

(9) $$\begin{array}{cc} \mathrm{generator}\;\mathrm{loss}=\; & \mathrm{BCE}\left(y_{\mathrm{true}},y_{\mathrm{pred}}\right)+\mathrm{MAE}\left(y_{\mathrm{true}},y_{\mathrm{pred}}\right)+ \end{array}$$

(10) $$\begin{array}{c} \;\;\;\;\;\;\;\;\;\mathrm{MSE}(y_{\mathrm{true}},y_{\mathrm{pred}})+0.1\times \mathrm{gradient}\;\mathrm{penalty}. \end{array}$$

The gradient penalty is calculated as follows:

(11) $$\mathrm{gradient}\;\mathrm{penalty}=\lambda E\left[(∥\nabla_{\hat{x}}D\left(\hat{x}\right){{∥}_{2}-1)}^{2}\right],$$

where $\hat{x}$ is the data sample and λ is a weighting term. The gradient penalty involves calculating the L2 norm of the gradient of the output with respect to its input. This regularization term ensures that the norm of the gradient is close to 1, promoting smoothness and stability in the synthetic data.

The discriminator model consists of a series of convolutional layers to distinguish between real and synthetic data, followed by a bidirectional LSTM layer, a spatiotemporal attention mechanism, and dense layers. The use of convolutional layers enables the discriminator to effectively capture local patterns in the data. The output layer of the discriminator uses a sigmoid activation function to provide a probability score that indicates the likelihood that a given sample is real.

In the following sections, we evaluate the quality of experiments and generate synthetic data using our proposed mechanisms and models.

## 4. Experiments

### 4.1. Data Description

**Figure 5:**
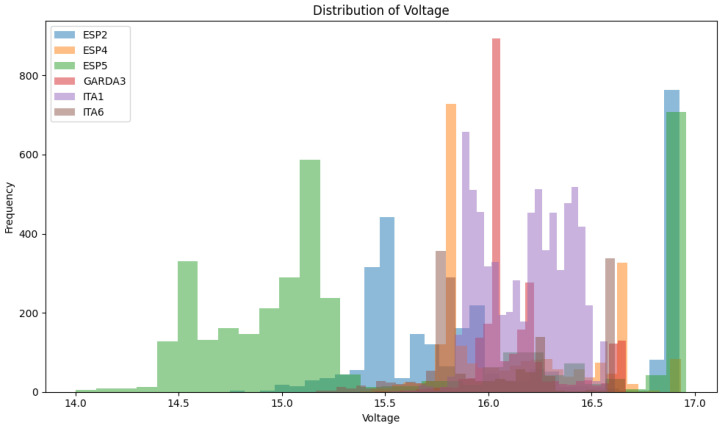
Distribution of each data variable over a total of 6 regions.

The dataset contains various parameters recorded during drone operation, such as date_time, latitude, longitude, altitude, EC, temp, DO, voltage, m0_current, m1_current, and heading. Latitude and longitude represent drone location information that is important to ensure diversity of flight paths, elevation represents drone height is important to obtain data at different altitudes, EC reflects the characteristics of the surrounding environment, temperature changes during a flight can affect drone performance, dissolved oxygen (DO) represents certain environmental conditions, voltage represents the power state of the drone that is critical to battery performance, m0_current, and m1_current represent the current usage of the drone motor, reflect the operational state, and heading shows the direction of the flight of drone, which is essential for generating different flight patterns. At this time, latitude, longitude, altitude, EC, temp, DO, voltage, and heading are important factors in drone operation. For instance, the detailed distribution of voltage data variables in each region used in our study is shown in Figure 5. Given the strong correlation between voltage and temperature, our attention mechanism addresses the rapidly changing latitude, longitude, altitude, and inverse correlation with EC while focusing on this relationship. Through this approach, we proceed with the generation of synthetic voltage data and evaluate the quality of the data.

### 4.2. Evaluation

#### 4.2.1. Experiment of Sensor Data Generation

We evaluated the data augmentation performance of the proposed *spatiotemporal* attention-based GAN to verify its feasibility as a sensor data augmentation technique aimed at alleviating computational load, power burden, and network burden in environments where data are collected using drones equipped with multiple sensors.

For each of the six datasets analyzed earlier, we generated values for each feature between sequentially listed timestamps with a constant stride. The stride represents the frequency of sensor data collection in a real environment. In the evaluation experiments, the stride was set to 2 to maximize the use of limited datasets. This means generating even-feature values between odd rows and odd-feature values between even rows. The stride can be adjusted, and increasing the stride value can enhance the data resolution. However, in such cases, it is advantageous for model performance to provide a broader range of input timestamps representing past and future data, allowing the model to generate data based on longer temporal relationships.

This evaluation was conducted using an Intel i5-11500 CPU @ 4.60 GHz, 32 GB RAM, and an Nvidia GeForce RTX 3060 Ti for training and data generation. The training conditions were set uniformly across all datasets, with 500 epochs and a batch size of 64. The focus is on generating realistic synthetic voltage data that accurately reflect the patterns observed in the original data. The evaluation process included visual and quantitative metrics to ensure a thorough assessment of the quality of synthetic data. Specifically, we compared the real and synthetic voltage data using the following methods:

1. **Visual Comparison:** To visually compare the real and synthetic data, we plotted the distribution of the actual voltage data alongside the synthetic data. This visual representation helps to identify whether the synthetic data capture the temporal dynamics and patterns present in the original dataset.
2. **Quantitative Metrics:** We employed several quantitative metrics to assess the similarity between the real and synthetic data. These metrics include:
  - **Mean Squared Error (MSE):** This metric measures the average squared difference between the real and synthetic voltage values. A lower MSE indicates that the synthetic data is close to the real data.
  - **Mean Absolute Error (MAE):** This metric calculates the average absolute difference between the real and synthetic values, providing another measure of accuracy.
  - **Pearson Correlation Coefficient (PCC):** PCC measures the linear correlation between the real and synthetic data, with values close to 1 indicating a strong positive correlation.
  - **Cosine Similarity:** This metric evaluates the cosine of the angle between two non-zero vectors, providing a measure of similarity that ranges from −1 to 1, where 1 indicates identical orientation.

**Figure 6:**
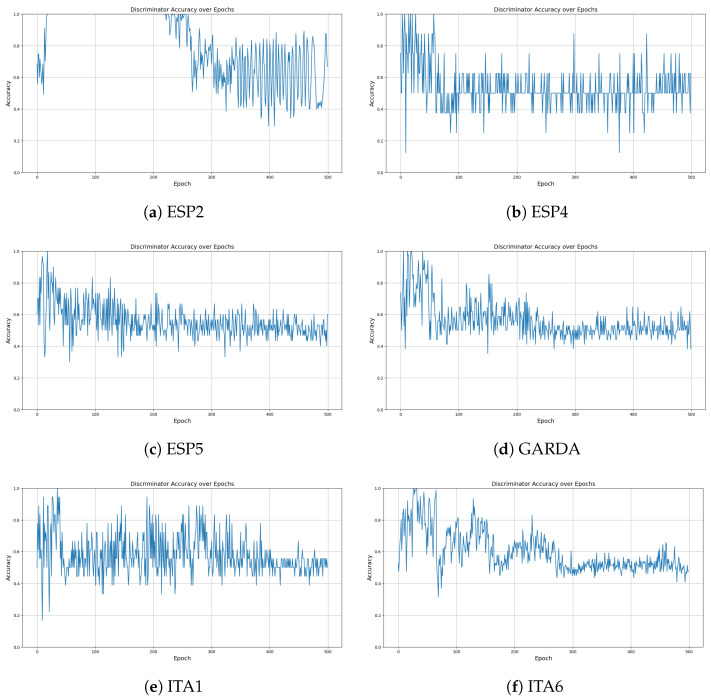
Discriminator training loss per datasets.

**Figure 7:**
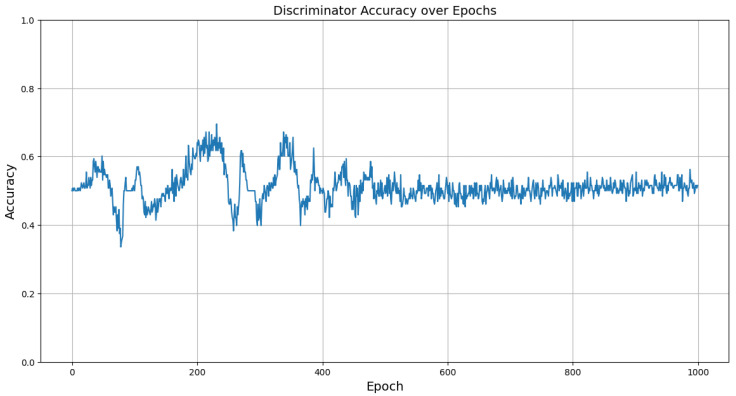
Accuracy by epoch of discriminator in the learning process.

**Figure 8:**
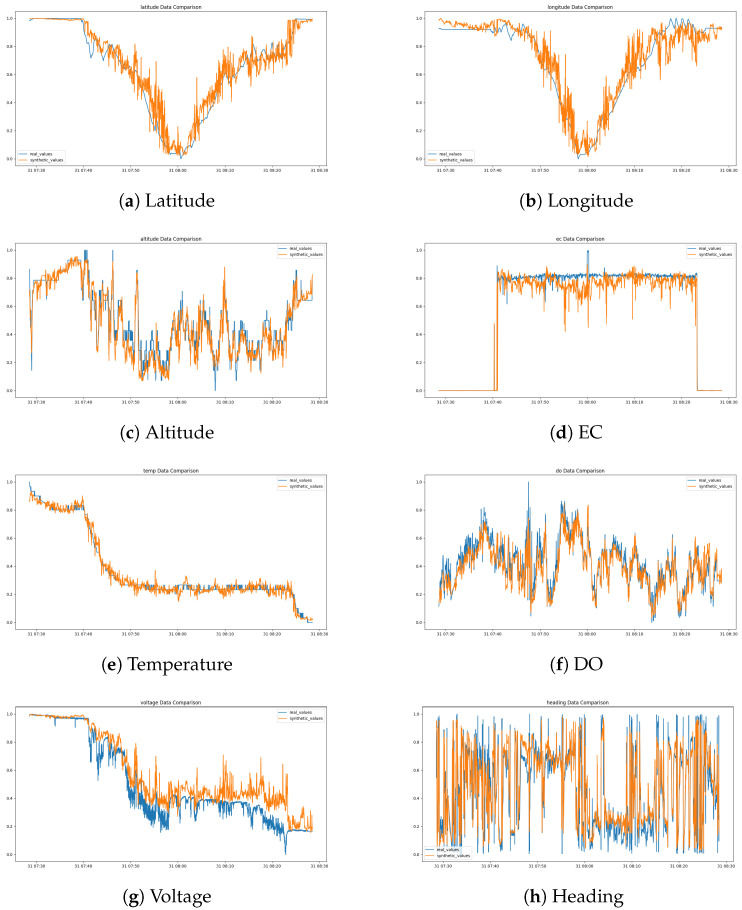
Visualization of Real Data and Synthetic Data. Orange is synthetic data and blue is original data.

**Figure 9:**
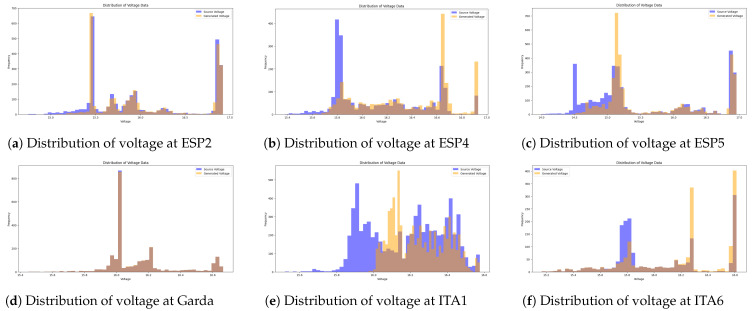
Comparison of drone voltage sensor data distribution in different regions. Orange is synthetic data and blue is original data.

Figure 6 shows the discriminator’s training accuracy per epoch for each dataset, demonstrating that each quickly converges to the target accuracy. This is a result of the newly applied attention mechanism and loss calculation technique. The accuracy here refers to the ability of the discriminator to classify real and synthetic data. An accuracy of around 50% indicates that the discriminator cannot differentiate between the two, signifying that the generator is producing realistic synthetic data. Therefore, achieving an accuracy near 50% is desirable, as it reflects the success of the generative model in mimicking real data as shown in Figure 7. Additionally, Figure 8 illustrates a comparison between the actual data and the synthetic data generated targeting the ESP5 dataset for each feature. The results indicate that the model has effectively learned the temporal relationships of the data to generate values at specific timestamps as shown in Figure 9. This finding is consistently reflected in Table 2, where various similarity metrics numerically compare the actual data and the synthetic data.

Through this evaluation, we confirmed the contribution of generating synthetic data that are almost indistinguishable from the actual values. This data augmentation technique is expected to improve the performance of autonomous drone operations and real-time monitoring systems, ultimately playing a crucial role in increasing data utilization in various application domains.

#### 4.2.2. Comparison with Other Models

In this work, we compare the proposed model with several state-of-the-art generative models, including DCT-GAN, MAD-GAN, LSTM-CNN, and 1D DCGAN, to compare it with GAN-based models that have recently shown better performance in handling complex time series data [25,44,45,46]. Each model was trained for 500 epochs for fair comparison. We chose these models based on their unique capabilities and effectiveness in processing multivariate time series data, which are important for application to sensor data augmentation.

Multivariate anomaly detection GAN (MAD-GAN) leverages multiple generators to capture different data modes, leveraging GAN’s descriptor loss as an additional metric to generate different samples [44]. At this time, the MAD-GAN used in this experiment uses three generators to learn different parts of the data distribution, respectively.

Diluted convolutional transformer-based GAN (DCT-GAN) is a model that combines convolution and transformer architecture to better capture complex patterns [45]. DCT-GAN enhances the ability to model complex time-series relationships by incorporating extended convolutions with zero padding in filter and transformer structures, including multi-head attention and feedforward networks.

LSTM-CNN GAN has been studied to be effective in solving vanishing gradient problems by incorporating bidirectional LSTM and 1D convolutional layers and capturing patterns from multivariate time series data [46].

Finally, 1D DCGAN has been studied for the diagnosis of electromechanical defects, and deep convolutional layers are known to be utilized to generate high-quality synthetic data for time series data [47].

Experimental results shown in Table 3 show that our model outperforms the control group in terms of accuracy and quality of the generated data. The four metrics listed in the table measure the similarity between vectors in different ways. MSE and MAE indicate higher similarity when closer to 0, while PCC and Cosine similarity indicate higher similarity when closer to 1. According to the table, the results derived using the proposed method demonstrate excellent similarity between real and synthetic data across all features. Although some models in the control group exhibit higher similarity in certain features compared to the proposed method, they show significantly lower similarity in other features. This indicates that the correlations between features have not been adequately learned. In contrast, the proposed method maintains harmony across the entire feature set while achieving high similarity.

Visual comparisons indicate that our model is more realistic and demonstrates its superiority in capturing the underlying patterns of sensor data by producing diverse samples. These results highlight the effectiveness of our approach in improving the reliability and robustness of sensor data augmentation tasks.

### 4.3. Use Case

**Figure 10:**
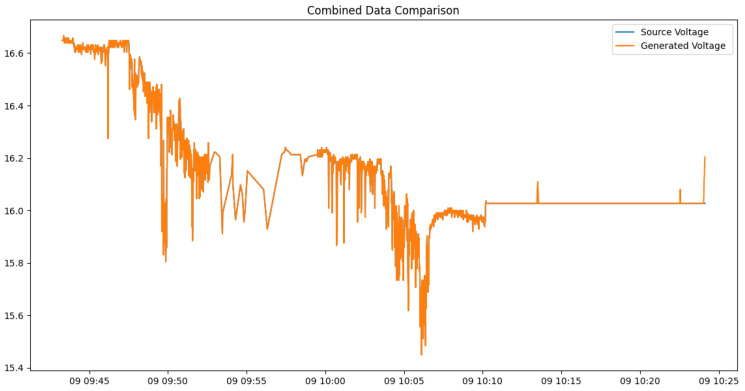
Comparison of original voltage data and synthetic voltage data.

The ability of spatial attention mechanisms to focus on important correlations between different sensor readings, especially voltage, temperature, latitude, longitude, and EC, emphasizes robustness and efficiency in accurately replicating real-world sensor data, including capturing outliers observed around 09:10:13 as shown in Figure 10, ensuring high fidelity in synthetic data generation. This high level of accuracy and realism in synthetic data is crucial for several following reasons.

Firstly, accurate synthetic battery data enable extensive testing and validation of drone systems under a wide range of conditions without the need for exhaustive real-world data collection. This is particularly beneficial in scenarios where obtaining real data is challenging, risky, or costly.

Secondly, synthetic data can be used to augment existing datasets, providing a more comprehensive training dataset for machine learning models used in drone navigation, control, and maintenance prediction. This leads to improved model robustness and generalization, allowing for better performance in real-world applications.

Moreover, the synthetic data help in stress testing and developing battery management systems. By analyzing how batteries perform under different simulated scenarios, we can develop more efficient algorithms for battery usage and charging, thereby extending the operational life and efficiency of drone fleets.

**Figure 11:**
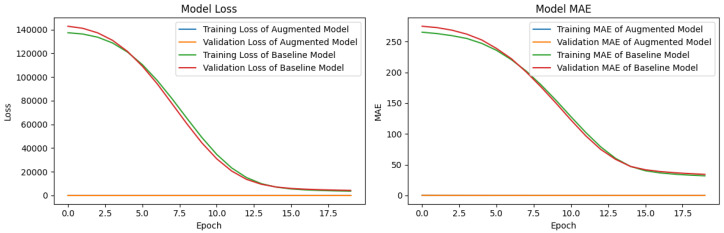
Comparison of baseline and augmented model with ESP2 dataset.

We demonstrate the effectiveness of the proposed augmented model in improving the performance of drone sensor data analysis, as shown in Figure 11. We implement a simple regression model based on a fully connected layer and compare the performance of the baseline model, which is a model using existing data, and the augmented model using augmented data. According to the results, the proposed augmented model shows almost overlapping curves in both loss and MAE for training and validation. This indicates that the values are on a much lower scale compared to the baseline model, implying that the augmented model learns significantly faster. This can be attributed to the use of the attention mechanism, which allows the model to achieve optimization quickly with fewer epochs. In summary, we leverage synthetic data generated from GANs using a novel spatiotemporal attention mechanism to improve both loss and MAE metrics over each epoch, effectively alleviating the limitations caused by less data.

These significant improvements highlight the utility of data augmentation in drone operational scenarios where collecting extensive real-world data can be impractical or expensive [48]. The ability of augmented models to generate high-quality synthetic data ensures a stronger training and validation process, leading to more accurate and reliable drone performance predictions [49]. Our research consequently suggests facilitating better mission planning, risk assessment, and management, ultimately improving the safety and efficiency of autonomous drone operations.

## 5. Conclusions

Drones have become essential tools across various industries due to their ability to provide real-time data and perform automated tasks. They are equipped with various sensors that enhance their functionality, but high-frequency data collection can overwhelm the onboard processing capabilities and network bandwidth, leading to inefficiencies. To address these challenges and maximize sensor data utility without increasing the physical and computational load on drones, our proposed GAN model with a novel attention mechanism demonstrates a robust solution for generating synthetic time series data. By leveraging *spatiotemporal* attention mechanism, we effectively capture both temporal dependencies and spatial correlations, ensuring the synthetic data closely mirrors real-world conditions. Our custom loss function further enhances the quality of the generated data, providing a reliable resource for training machine learning models in drone power management. We validate the feasibility of the proposed technique through performance evaluations using various assessment metrics and comparison models.

In future work, it is necessary to generally verify the performance of our proposed generative model in scenarios utilizing additional drone datasets of various environments and operating conditions. In addition, as part of our future work, we plan to universally evaluate our model’s performance by comparing it to various generative models in addition to GAN. This aims to ensure the wide applicability and robustness of the synthetic data generation approach proposed in this paper.

## Figures and Tables

**Table 1:** Data Description of Drone Sensor.

Campaign	Location	Date	Duration (min)	Samples
ESP2	River Ter, Torrello, Barcelona, Spain	31 March 2017	47	2814
ESP4	Pantà de Sau Reservoir, Barcelona, Spain	30 March 2017	39	2374
ESP5	Bayou Ter River, Barcelona, Spain	31 March 2017	60	3601
GARDA3	Lake Gardena, Verona, Italy	9 May 2017	40	2451
ITA1	Atlantide Fishing Site, Verona, Italy	20 April 2017	121	7243
ITA6	Atlantide Fishing Site, Verona, Italy	7 March 2017	28	1704

**Table 2:** Similarity Results of Spatiotemporal-Attention GAN on Drone Sensor Data.

Model	Metric	Latitude	Longitude	Altitude	EC	Temp	DO	Voltage	Heading
Spatiotemporal -Attention GAN	MSE	0.0061	0.0071	0.0054	0.0040	0.0009	0.0042	0.0120	0.0172
	MAE	0.0553	0.0633	0.0564	0.0371	0.0231	0.0524	0.0801	0.0881
	PCC	0.9722	0.9614	0.9578	0.9894	0.9928	0.9406	0.9669	0.8831
	Cosine	0.9944	0.9942	0.9915	0.9969	0.9977	0.9922	0.9878	0.9715

**Table 3:** Comparison with Other Models on Drone Sensor Data.

Model	Metric	Latitude	Longitude	Altitude	EC	Temp	DO	Voltage	Heading
DCGAN	MSE	0.0000	0.0000	12.7468	93,772.9686	1.8734	0.2295	0.7258	25,369.4697
	MAE	0.0004	0.0015	2.8572	239.6136	1.1365	0.3903	0.7071	127.7668
	PCC	0.0086	0.0010	0.0370	−0.0010	−0.0224	−0.0067	−0.0179	−0.0161
	Cosine	1.0000	1.0000	1.0000	0.5387	0.9931	0.9978	0.9986	0.7159
LSTM-CNN GAN	MSE	0.0000	0.0000	8.3709	69,270.8039	5.6076	0.0917	0.9790	22,981.1240
	MAE	0.0004	0.0013	2.3484	221.3266	1.7811	0.2630	0.7669	122.8213
	PCC	0.0115	0.0120	0.0060	−0.0167	0.0112	0.0148	0.0064	−0.0171
	Cosine	1.0000	1.0000	1.0000	0.5779	0.9853	0.9997	0.9981	0.7801
MAD-GAN	MSE	0.0000	0.0000	19.3150	127,297.3289	2.0403	0.2860	0.8311	38,136.2132
	MAE	0.0004	0.0016	3.7649	278.3726	1.1939	0.4398	0.7502	158.1357
	PCC	0.0185	−0.0380	−0.0195	−0.0256	0.0176	−0.0400	0.0166	0.0067
	Cosine	1.0000	1.0000	1.0000	0.3699	0.9929	0.9973	0.9985	0.6877
DCT-GAN	MSE	0.0000	0.0000	6.2528	77,151.9267	1.6957	0.0799	0.7473	20,965.9737
	MAE	0.0004	0.0015	1.9984	217.4320	1.0775	0.2253	0.7229	118.0895
	PCC	−0.0055	0.0085	−0.0162	−0.0164	0.0183	0.0047	0.0044	−0.0142
	Cosine	1.0000	1.0000	1.0000	0.6205	0.9942	0.9994	0.9988	0.7941
Spatiotemporal -Attention GAN (Proposed)	MSE	0.0061	0.0071	0.0054	0.0040	0.0009	0.0042	0.0120	0.0172
	MAE	0.0553	0.0633	0.0564	0.0371	0.0231	0.0524	0.0801	0.0881
	PCC	0.9722	0.9614	0.9578	0.9894	0.9928	0.9406	0.9669	0.8831
	Cosine	0.9944	0.9942	0.9915	0.9969	0.9977	0.9922	0.9878	0.9715

## Data Availability

The data presented in this study are available on request from the corresponding author.
